# Peripheral Blood Occludin Level as a Biomarker for Perioperative Cerebral Edema in Patients with Brain Tumors

**DOI:** 10.1155/2020/8813535

**Published:** 2020-08-19

**Authors:** Shuhai Shi, Jingli Cheng, Chunyang Zhang, Tao Liang, Yunxin Zhang, Yongxing Sun, Ying Zhao, Weili Li, Baoguo Wang

**Affiliations:** ^1^Department of Critical Care Medicine, Sanbo Brain Hospital, Capital Medical University, Beijing 100093, China; ^2^Department of Neurosurgery, First Affiliated Hospital of Baotou Medical College, Baotou 014010, Inner Mongolia Autonomous Region, China; ^3^Department of Anesthesiology, Sanbo Brain Hospital, Capital Medical University, Beijing 100093, China; ^4^Department of Neurology, Xuanwu Hospital, Capital Medical University, Beijing 100053, China

## Abstract

**Objective:**

Cerebral edema is a common complication of brain tumors in the perioperative period. However, there is currently no reliable and convenient method to evaluate the extent of brain edema. The objective is to explore the effectiveness of serum occludin on predicting the extent of perioperative brain edema and outcome in patients with brain tumors.

**Methods:**

This prospective study enrolled 55 patients with brain tumors and 24 healthy controls in Sanbo Brain Hospital from June 2019 through November 2019. Serum occludin levels were measured preoperatively and on postoperative day 1. Peritumoral edema was assessed preoperatively using MRI. Pericavity brain edema on postoperative day 1 was evaluated using CT.

**Results:**

Compared with healthy controls, the serum occludin level was higher in patients with brain tumors both preoperatively and postoperatively (*P* < 0.001). The serum occludin level correlated positively with the degree of brain edema preoperatively (*r* = 0.78, *P* < 0.001) and postoperatively (*r* = 0.59, *P* < 0.001). At an optimal cutoff of 3.015 ng/mL, the preoperative serum occludin level discriminated between mild and severe preoperative brain edema with a sensitivity of 90.48% and specificity of 84.62%. At an optimal cutoff value of 3.033 ng/mL, the postoperative serum occludin level distinguished between mild and severe postoperative brain edema with a sensitivity of 97.30% and specificity of 55.56%.

**Conclusions:**

The serum occludin level is associated with cerebral edema and could potentially be used as a biomarker for perioperative cerebral edema. This trial is registered with ChiCTR1900023742.

## 1. Introduction

Damage to the blood-brain barrier (BBB) in patients with brain tumors can cause cerebral edema or hemorrhage. Brain edema, as a major factor that governs clinical management, is responsible for clinical symptoms such as neurological deficit and intracranial hypertension. The severity of perioperative brain edema and the occurrence of postoperative hemorrhage in patients with brain tumors are directly related to prognosis and risk of death [1]. Computed tomography (CT) and magnetic resonance imaging (MRI) can be used to indirectly evaluate the extent of brain edema and hemorrhage in patients with brain tumors. However, these imaging techniques are inconvenient and time-consuming and so cannot be used to screen for rapid deteriorations in the postoperative condition of a patient in the intensive care unit (ICU). Therefore, it is vital that a reliable biomarker be identified that reflects the degree of cerebral edema and risk of cerebral hemorrhage in the perioperative period, as this will facilitate the prevention of and/or timely intervention for cerebral edema or hemorrhage after surgery for craniocerebral tumors.

The expressions of several proteins in brain tissue, including N-cadherin, *β*-catenin, aquaporin-4, delta-like protein-4, matrix metallopeptidase- (MMP) 9, and vascular endothelial growth factor, are associated with the occurrence of peritumoral brain edema (PTBE) [2–5]. However, few studies have examined the relationships between serum proteins and PTBE.

Tight junction proteins such as claudin-5 and occludin are key structural components of the BBB [6, 7]. Occludin and claudin-5 form tight junction protein structures that seal the gap between the endothelial cells of the BBB and thus are responsible for maintaining the integrity of the BBB [8]. Since occludin expression alone does not result in tight junction formation [9], it is likely that claudin-5 forms the primary structure of the tight junction with occludin acting as an additional supporting structure [10]. This may make occludin vulnerable to release into the bloodstream following damage to the BBB. PTBE formation is widely accepted to be vasogenic brain edema due to the damage of BBB. There is some evidence that decreased expressions of occludin and claudin-5 in brain tissue are related to PTBE [11, 12], suggesting that brain edema can arise if occludin and claudin-5 are lost from the BBB. Our recent studies have confirmed that degraded tight junction protein fragments are released into the blood and that their levels in the blood are closely related to the degree of BBB damage after cerebral ischemia [13, 14]. This suggests that occludin could potentially be used as a biomarker to evaluate damage to the BBB. However, no previous investigations have reported whether occludin is degraded and released into the bloodstream because of damage to the BBB by a brain tumor or brain surgery.

It is widely recognized that patients with brain tumors can exhibit damage to their BBB both before and after surgery. Therefore, we hypothesized that the occludin level in peripheral blood might serve as a biomarker for cerebral edema due to BBB damage in patients with brain tumors. To the best of our knowledge, there are currently no studies describing the association between peripheral blood occludin levels and the extent of PTBE or postoperative pericavity edema in patients with brain tumors. Therefore, the aim of the present study was to investigate the relationships between the serum occludin level and the extent of PTBE before surgery and the extent of pericavity brain edema after surgery in patients with brain tumors.

## 2. Material and Methods

### 2.1. Study Design and Participants

This prospective study enrolled consecutive patients with brain tumors scheduled for surgery at the Department of Neurosurgery, Sanbo Brain Hospital, Capital Medical University, between June 1st, 2019, and November 1st, 2019. A control group of healthy people was also enrolled at the Physical Examination Center, Sanbo Brain Hospital, during the same period. The inclusion criteria for patients with brain tumors were as follows: (1) primary brain tumor confirmed by imaging investigations such as CT and MRI, (2) met the indications for surgical treatment and scheduled to undergo surgery, (3) aged 18–80 years old, (4) had not received radiotherapy or chemotherapy before surgery, and (5) provided informed consent for inclusion in the study. The exclusion criteria were other diseases that might lead to the destruction of tight junction proteins between vascular endothelial cells, including (1) severe hepatorenal disease, (2) severe gastrointestinal system disease, (3) peripheral vascular disease, and (4) autoimmune disease. The healthy people included as a control group were randomly selected from persons undergoing routine physical examinations at Sanbo Brain Hospital. Patients with craniopharyngioma or hypophysoma were treated at a dose of 250 mg methylprednisolone, and the other brain tumor patients received 5 mL/kg 20% mannitol intravenously for 3 days after operation. The study was approved by the Ethics Committee of Sanbo Brain Hospital, and all patients provided informed written consent for inclusion in the study.

### 2.2. Measurement of Serum Occludin Levels

Peripheral venous blood samples (4 mL) were obtained from patients with brain tumors at admission (i.e., preoperatively) and approximately 24 hours (range, 20–28 hours) after surgery. Blood samples from healthy subjects were taken during their visit to the physical examination center. Serum (100 *μ*L) was separated from each blood sample, and the level of occludin in the serum was measured using a commercially available enzyme-linked immunosorbent assay (ELISA) kit for human samples (Occludin: USCN, Wuhan, China).

### 2.3. Measurement of PTBE before Surgery

**Figure 1:**
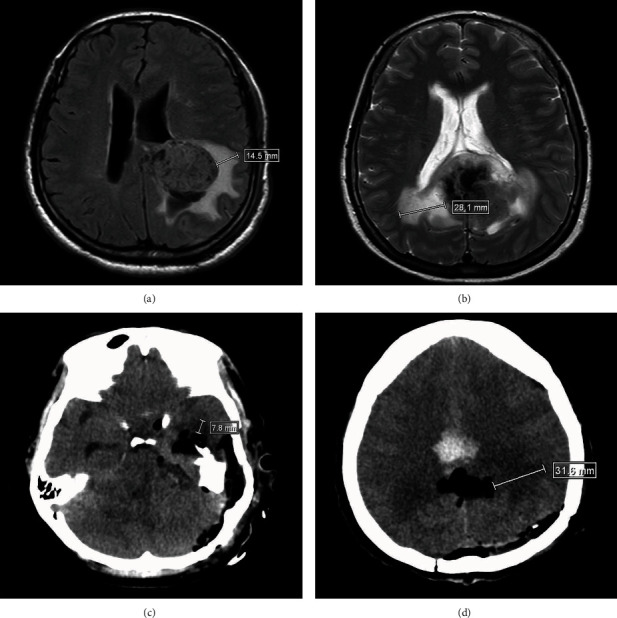
Evaluation of peritumoral brain edema (PTBE) and pericavity brain edema by magnetic resonance imaging (MRI) and computed tomography (CT), respectively. (a) MRI scan showing mild PTBE before surgery. (b) MRI scan showing severe PTBE before surgery. (c) CT scan showing mild brain edema around the cavity after surgery. (d) CT scan showing severe brain edema around the cavity after surgery.

PTBE was evaluated by 1.5T MRI (Achieva 1.5T, Philips, Amsterdam, Netherlands) before surgery (Figures 1(a) and 1(b)). The imaging sequences used in this study included axial T2-weighted sequences, fluid-attenuated inversion recovery (FLAIR) sequences, and axial and coronal T1-weighted spin-echo sequences before and after intravenous injection of the contrast agent (gadopentetate dimeglumine; 0.1 mg/kg body weight). All images were analyzed digitally using PACS workstations. Enhanced T1-weighted images were used to determine the tumor boundary, and these maps were then compared with the respective T2-weighted images or FLAIR sequences. The degree of PTBE was determined by measuring the vertical distance from the outer edge of the maximal edema zone to the tumor boundary. PTBE was graded according to the Steinhoff classification [15] as follows: 0, no edema; I, PTBE limited to 2 cm; II, PTBE limited to half the hemisphere; and III, PTBE extending to more than half the hemisphere. For the analysis, mild edema was defined as Steinhoff grade 0 or I, and severe edema was defined as Steinhoff grade II or III. Determination of the Steinhoff grade was made independently by two clinicians who were deputy directors of imaging and had more than 5 years of work experience, and any disagreements were resolved by discussion.

### 2.4. Measurement of Pericavity Edema after Surgery

Pericavity brain edema was evaluated by CT (Brilliance 64, Philips) at approximately 24 hours (range, 20–28 hours) after surgery (Figures 1(c) and 1(d)). The degree of pericavity edema was measured as the vertical distance from the outer edge of the maximal edema zone to the cavity boundary after surgery. Evaluation of the degree of edema was the same as that for PTBE (*vide supra*).

### 2.5. Measurement of Neurological Function and Related Complications

To further analyze the relationships between the serum occludin level and the neurological function and related complications, the National Institutes of Health Stroke Scale (NIHSS) and Glasgow Coma Scale (GCS) were administered at admission and at around 24 hours (range, 20–28 hours) after surgery. Furthermore, information regarding the occurrence of intracranial hemorrhage was collected at approximately 24 hours (range, 20–28 hours) after surgery [16].

### 2.6. Statistical Analysis

The data were analyzed using SPSS 22.0 (IBM Corp., Armonk, NY, USA). All continuous data were confirmed to be normally distributed and so are presented as mean ± standard deviation (SD). One-way analysis of variance was used to compare serum levels of occludin between groups. NIHSS and GCS scores were compared between groups using Student's *t*-test. Categorical data are presented as *n* (%) and were compared between groups using the chi-squared test or Fisher's exact test. The relationship between the extent of the brain edema (vertical distance from the outer edge of the maximal edema zone to the tumor/cavity boundary) and the serum occludin level was assessed by calculation of Pearson's correlation coefficient (*r*). Receiver operating characteristic (ROC) curve analysis, with calculation of the area under the curve (AUC), was used to evaluate the ability of the serum occludin level to distinguish between mild and severe edema. The optical cutoff for the serum occludin level was established by calculation of the Youden index, and sensitivity and specificity values were determined. Statistical significance was defined as a two-tailed *P* value < 0.05.

## 3. Results

### 3.1. Baseline Clinical Characteristics of the Study Participants

A total of 55 patients with brain tumors and 24 healthy people were enrolled in this study. The group of patients with brain tumors and control group of healthy people exhibited no significant differences in mean age (47.42 ± 15.72 years vs. 40.63 ± 14.42 years) or gender (56.4% female vs. 51.2% female; Table 1). Additional clinical data for the patients with brain tumors are shown in Table 1.

### 3.2. Comparison of Serum Occludin Levels between Patients with Brain Tumors and Healthy Volunteers

**Figure 2:**
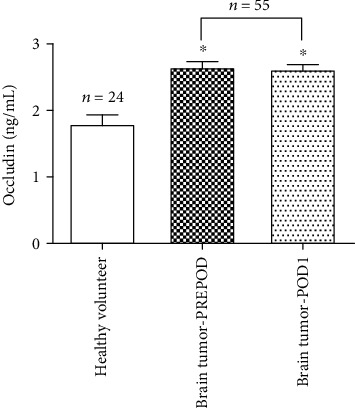
Comparison of serum occludin levels between patients with brain tumors and healthy volunteers. Compared with healthy volunteers (*n* = 24), serum occludin levels in patients with brain tumors (*n* = 55) were significantly higher both before surgery (Brain tumor-PREPOD) and 1 day after surgery (Brain tumor-POD1). Data are expressed as mean ± standard deviation. ^∗^*P* < 0.05 versus the healthy volunteer group.

There was no significant difference between the preoperative and postoperative serum occludin levels in patients with brain tumors. However, both the preoperative and postoperative serum occludin levels in patients with brain tumors were significantly higher than the levels in healthy people (Figure 2). These results suggest that brain tumors may induce damage to the BBB, leading to the release of occludin into the bloodstream.

### 3.3. Analysis of the Relationship between the Preoperative Serum Occludin Level and the Extent of the Preoperative PTBE and Tumor Diameter

**Figure 3:**
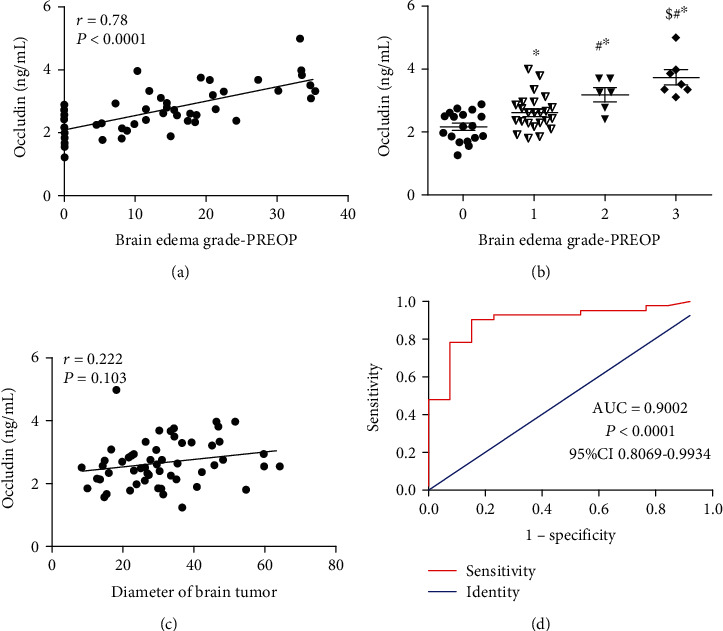
The relationship between the preoperative serum occludin level and the peritumoral brain edema (PTBE) before surgery. (a) Pearson's correlation analysis showing that the preoperative serum occludin level was significantly positively correlated with the extent of the preoperative PTBE as measured by the vertical distance from the outer edge of the maximal edema zone to the tumor boundary (*n* = 55). (b) Preoperative serum occludin levels for the various Steinhoff grades of PTBE. Data are presented as mean ± standard deviation (grade 0, *n* = 18; grade I, *n* = 24; grade II, *n* = 6; and grade III, *n* = 7). ^∗^*P* < 0.05 vs. grade 0; ^#^*P* < 0.05 vs. grade I; and ^$^*P* < 0.05 versus grade II. (c) Pearson's correlation analysis showing that the preoperative serum occludin level was not significantly correlated with the tumor diameter. (d) Receiver operating characteristic curve for the ability of the serum occludin level to distinguish between mild and severe PTBE. AUC: area under the curve; CI: confidence interval.

Pearson's correlation analysis revealed that the preoperative serum occludin level was significantly positively correlated with the extent of the preoperative PTBE as measured by the vertical distance from the outer edge of the maximal edema zone to the tumor boundary (*r* = 0.78, *P* < 0.0001; Figure 3(a)). Furthermore, the preoperative serum occludin level was also significantly correlated with the degree of preoperative PTBE as measured by the Steinhoff grade (*P* < 0.05 for all pairwise comparisons; Figure 3(b)). However, the preoperative serum occludin level was not significantly correlated with the diameter of the tumor (*r* = 0.222; *P* = 0.103; Figure 3(c)).

### 3.4. ROC Curve Analysis of the Ability of the Preoperative Serum Occludin Level to Predict the Severity of Preoperative PTBE

ROC curve analysis indicated that the preoperative serum occludin level showed excellent ability to distinguish between mild and severe PTBE, with an AUC value of 0.9002 (95% confidence interval, 0.8069–0.9934; *P* < 0.0001; Figure 3(d)). At an optimal cutoff value for the preoperative serum occludin level of 3.015 ng/mL, the sensitivity was 90.48% and the specificity was 84.62%. These results suggest that the preoperative serum occludin level could potentially be used to reflect the degree of PTBE before surgery.

### 3.5. Analysis of the Relationship between the Postoperative Serum Occludin Level and the Extent of the Pericavity Edema and Tumor Diameter

**Figure 4:**
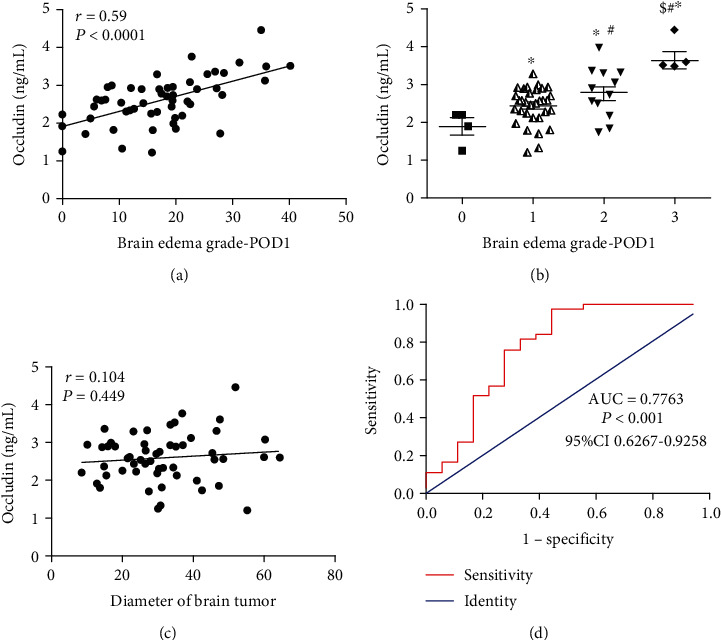
The relationship between the postoperative serum occludin level and the pericavity brain edema after surgery. (a) Pearson's correlation analysis indicating that the postoperative serum occludin level was significantly positively correlated with the extent of the postoperative pericavity edema as measured by the vertical distance from the outer edge of the maximal edema zone to the cavity boundary (*n* = 55). (b) Postoperative serum occludin levels for the various Steinhoff grades of pericavity edema. Data are presented as mean ± standard deviation (grade 0, *n* = 4; grade I, *n* = 33; grade II, *n* = 13; and grade III, *n* = 5). ^∗^*P* < 0.05 vs. grade 0; ^#^*P* < 0.05 vs. grade I; and ^$^*P* < 0.05 versus grade II. (c) Pearson's correlation analysis indicating that the postoperative serum occludin level was not significantly correlated with the tumor diameter. (d) Receiver operating characteristic curve for the ability of the postoperative serum occludin level to distinguish between mild and severe pericavity edema. AUC: area under the curve; CI: confidence interval.

Pearson's correlation analysis indicated that the postoperative serum occludin level was significantly positively correlated with the extent of the postoperative pericavity edema as measured by the vertical distance from the outer edge of the maximal edema zone to the cavity boundary (*r* = 0.590, *P* < 0.0001; Figure 4(a)). Similarly, the postoperative serum occludin level was significantly related to the degree of the postoperative pericavity edema as measured by the Steinhoff grade (*P* < 0.05 for all pairwise comparisons; Figure 4(b)). The postoperative serum occludin level was not significantly correlated with the tumor diameter (*r* = 0.104, *P* = 0.449; Figure 4(c)).

### 3.6. ROC Curve Analysis of the Ability of the Postoperative Serum Occludin Level to Predict the Severity of Postoperative Pericavity Edema

ROC curve analysis demonstrated that the postoperative serum occludin level had good ability to discriminate between mild and severe pericavity edema, with an AUC value of 0.7763 (95% confidence interval, 0.6267–0.9258; *P* < 0.0001; Figure 4(d)). At an optimal cutoff value for the postoperative serum occludin level of 3.033 ng/mL, the sensitivity was 97.30% and the specificity was 55.56%.

### 3.7. Relationship between the Preoperative Serum Occludin Level and the Clinical Outcomes

**Figure 5:**
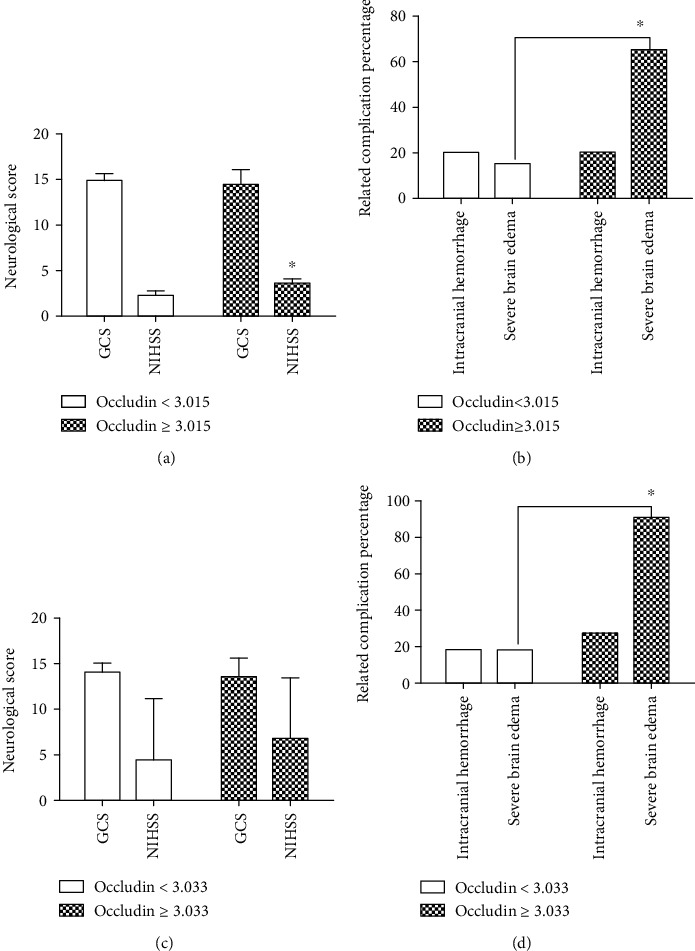
The relationship between the serum occludin level and the clinical outcomes in patients with brain tumors. (a) Comparison of National Institutes of Health Stroke Scale (NIHSS) and Glasgow Coma Scale (GCS) scores at admission between patients with a preoperative occludin level < 3.015 ng/mL (*n* = 40) and those with a preoperative occludin level ≥ 3.015 ng/mL (*n* = 15). (b) Comparison of the incidences of preoperative severe brain edema and intracranial hemorrhage between patients with a preoperative occludin level < 3.015 ng/mL (*n* = 40) and those with a preoperative occludin level ≥ 3.015 ng/mL (*n* = 15). (c) Comparison of NIHSS and GCS scores at 1 day after surgery between patients with a postoperative occludin level < 3.033 ng/mL (*n* = 44) and those with a postoperative occludin level ≥ 3.033 ng/mL (*n* = 11). (d) Comparison of the incidences of postoperative severe brain edema and intracranial hemorrhage between patients with a postoperative occludin level < 3.033 ng/mL (*n* = 44) and those with a postoperative occludin level ≥ 3.033 ng/mL (*n* = 11).

To examine whether the preoperative serum occludin level might be related to clinical outcomes, patients were divided into two groups according to the optimal cutoff value for the preoperative occludin level (3.015 ng/mL). Compared with patients with a preoperative serum occludin level < 3.015 ng/mL, those with an occludin level ≥ 3.015 ng/mL had a significantly higher NIHSS score at admission (3.60 ± 0.48 vs. 2.31 ± 0.42; *P* < 0.001; Figure 5(a)) and significantly higher incidence of severe PTBE at admission (65.3% vs. 15.2%; *P* < 0.001; Figure 5(b)) but similar GCS score at admission (14.47 ± 1.60 vs. 14.85 ± 0.70; *P* = 0.219; Figure 5(a)) and similar incidence of intracranial hemorrhage at 1 day after surgery (20.0% vs. 20.0%; *P* = 1.000; Figure 5(b)).

### 3.8. Relationship between the Postoperative Serum Occludin Level and the Clinical Outcomes

The relationship between the postoperative serum occludin level and the clinical outcomes was also assessed. Compared with patients with a postoperative serum occludin level < 3.033 ng/mL (the optimal cutoff value), those with an occludin level ≥ 3.033 ng/mL had a significantly higher incidence of severe pericavity edema at 1 day after surgery (90.9% vs. 18.2%; *P* < 0.001) but a similar NIHSS score (6.82 ± 6.64 vs. 4.52 ± 6.66; *P* = 0.306), GCS score (13.55 ± 2.07 vs. 14.09 ± 1.09; *P* = 0.407), and incidence of intracranial hemorrhage (27.3% vs. 18.2%; *P* = 0.800) at 1 day after surgery (Figures 5(c) and 5(d)).

## 4. Discussion

The present report demonstrates that patients with brain tumors exhibit perioperative changes in the serum level of a protein that contributes to the structure and function of the BBB. Importantly, this study revealed a relationship between the serum level of occludin and the severity of brain edema in patients with brain tumors. Previous investigations have confirmed that peritumoral brain edema is vascular edema caused by damage to the BBB [17–19]. Occludin is an important component of tight junctions that constitute the BBB. Previous animal experiments have reported that cerebral ischemia results in the activation of MMP-2 and MMP-9 in brain tissue, leading to the degradation of occludin and destruction of the BBB [20, 21]. In addition, a study of intracerebral hemorrhage has revealed that circulating tight junctions could be considered the potential biomarkers reflecting the integrity of the BBB in intracranial hemorrhage [22]. The present study found that the serum occludin level was increased in patients with brain tumors as compared with healthy controls, suggesting that tumor-induced damage to the BBB during the perioperative period may lead to the loss of occludin from the BBB. Furthermore, we demonstrated that the serum occludin level correlated well with the degree of brain edema and could be used to predict the severity of perioperative brain edema in patients with brain tumors.

We found that the serum level of occludin was significantly higher in patients with brain tumors, both before and after surgery, than in healthy people. These results suggest that damage to the BBB occurs in patients with brain tumors, leading to the release of degraded occludin fragments into the blood and thus an increase in the serum level of occludin. Notably, the patients included in this study had a broad range of tumor types located in different parts of the brain, suggesting that damage to the BBB and degradation/release of occludin may be a common consequence of many different types of brain tumors, irrespective of the mechanism by which the BBB is damaged. Therefore, our study has identified a novel circulating biomarker that may be beneficial for the detection or/and monitoring of BBB damage during the perioperative period in patients with brain tumors. Of course, future studies will need to establish whether the performance of the serum occludin level as a clinical biomarker varies between different pathological types of tumor.

Interestingly, we found a positive correlation between the peritumoral or pericavity brain edema and the level of serum occludin, with a higher occludin level associated with more severe brain edema. Notably, the serum occludin level was related only to the degree of brain edema and not to the size of the tumor. Vascular brain edema is a well-recognized feature of BBB damage, which likely leads to the release of degraded occludin. Previous studies have shown that peritumoral brain edema is associated with a low content of occludin in the tumor [23, 24], but it has not been clarified whether the low content of occludin in the BBB of brain tumors is mainly due to degradation or low expression of occludin. Our observation of an elevated level of serum occludin suggests that a low content of occludin within a tumor may, at least in part, be due to the degradation of occludin rather than downregulated expression. There is also some evidence that matrix metalloproteinases (MMPs) are considered to be the key to mediate the opening of the blood-brain barrier [25] and highly expressed in brain tumors which can be activated by the upregulation of the beta-catenin pathway [26]. They may play an important role in the destruction of the blood-brain barrier. Further studies are needed to clarify the mechanisms underlying BBB damage and alterations in brain tumor levels of occludin.

Depending on the type of the brain tumor, the development of brain edema can involve a variety of factors including glioma cells, vascular endothelial cells, neuroglial cells, microglial cells [27, 28], and cyclooxygenase-2 [29]. Our study found that the serum occludin level correlated well with the grade of brain edema, suggesting that damage to occludin in the BBB may be a common molecular mechanism underlying the development of brain edema in patients with brain tumors. Some studies of glioma have shown that MMP expression or activation is related to the downregulation of occludin in the BBB, which leads to destruction of the BBB [30, 31]. It is possible that MMP activation may be a major mechanism by which degradation of occludin and damage to the BBB occur in patients with brain tumors. However, further research is needed to clarify the relevant molecular mechanisms in humans.

This study utilized ROC curve analysis to evaluate the ability of the preoperative and postoperative serum occludin levels to distinguish between mild and severe edema. The preoperative and postoperative serum occludin levels showed excellent (AUC value of 0.9002) and good (AUC value of 0.7763) discriminatory ability, respectively. At an optimal cutoff value of 3.015 ng/mL, the preoperative serum occludin level discriminated between mild and severe preoperative brain edema with a high sensitivity (90.48%) and specificity (84.62%). At an optimal cutoff value of 3.033 ng/mL, the postoperative serum occludin level also distinguished between mild and severe postoperative brain edema with a high sensitivity (97.30%), although the specificity was somewhat lower (55.56%). Taken together, the findings of the ROC curve analysis suggest that the serum occludin level has the potential to be a novel biomarker for perioperative brain edema in patients with brain tumors.

Brain edema may be an important factor affecting the neurological function of patients with brain tumors. This study found that patients with a high serum occludin level before surgery also had a higher preoperative NIHSS score, which may be related to more severe damage to the BBB. However, although the group of patients with elevated serum occludin levels after surgery had more severe brain edema, their postoperative NIHSS score did not differ from that for patients with lower occludin levels. This apparent discrepancy may be due to the neurological functions of these patients being more affected by other surgery-related factors. We also found no difference in the incidence of postoperative cerebral hemorrhage between patients with high and low preoperative or postoperative levels of occludin. This latter finding suggests that postoperative acute cerebral hemorrhage may be primarily related to surgical factors (such as damage to microvessels or incomplete hemostasis) rather than BBB injury.

This study has some limitations. First, the sample size was not large, and an extensive range of tumor pathological types was included. Further large-scale clinical studies are needed to validate our results and perform subgroup analyses based on different brain tumor types. Second, we did not perform long-term follow-up to evaluate the prognosis of the patients and explore whether outcomes were related to the serum occludin level.

## 5. Conclusion

The perioperative serum occludin level is associated with the severity of peritumoral/pericavity edema in patients with brain tumors. The serum occludin level could potentially be used as a biomarker for perioperative cerebral edema in patients with brain tumors. Further large-scale clinical studies are needed to validate our results and perform subgroup analyses based on different brain tumor types.

## Figures and Tables

**Table 1:** Baseline clinical characteristics of the study participants.

Characteristic	Patients with brain tumors (*n* = 55)	Healthy volunteers (*n* = 24)	*P*
Age (years)	47.42 ± 15.72	40.63 ± 14.42	0.074
Female	31 (56.4%)	13 (51.2%)	0.857
Tumor location			
Frontal lobe	15 (27.3%)	N/A	N/A
Temporal lobe	14 (25.5%)	N/A	N/A
Occipital lobe	6 (10.9%)	N/A	N/A
Parietal lobe	3 (5.5%)	N/A	N/A
Sella region	6 (10.9%)	N/A	N/A
Cerebellum	4 (7.3%)	N/A	N/A
Brain stem	5 (9.1%)	N/A	N/A
Basal ganglia	2 (3.6%)	N/A	N/A
Tumor pathology			
Glioma	22 (40.0%)	N/A	N/A
Meningioma	12 (21.8%)	N/A	N/A
Neurilemmoma	7 (12.7%)	N/A	N/A
Hypophysoma	4 (7.3%)	N/A	N/A
Hemangioma	4 (7.3%)	N/A	N/A
Germinoma	2 (3.6%)	N/A	N/A
Craniopharyngioma	2 (3.6%)	N/A	N/A
Chordoma	1 (1.8%)	N/A	N/A
Cholesteatoma	1 (1.8%)	N/A	N/A

Data are presented as mean ± standard deviation or *n* (%). N/A: not applicable.

## Data Availability

The results of our study supporting the findings are included within this paper. In order to protect patient privacy, the personal data supporting the findings of this study are restricted by the Ethics Committee of Sanbo Brain Hospital, Capital Medical University. Data are available to researchers who meet the criteria for access to confidential data from the corresponding author.
